# Right Ventricular Function in Arrhythmogenic Right Ventricular Cardiomyopathy: Potential Value of Strain Echocardiography

**DOI:** 10.3390/jcm13030717

**Published:** 2024-01-26

**Authors:** Caroline Løkke Bjerregaard, Tor Biering-Sørensen, Kristoffer Grundtvig Skaarup, Morten Sengeløv, Mats Christian Højbjerg Lassen, Niklas Dyrby Johansen, Flemming Javier Olsen

**Affiliations:** 1 Department of Cardiology, Copenhagen University Hospital—Herlev and Gentofte, 2900 Hellerup, Denmark; bjerregaard.caroline@gmail.com (C.L.B.);; 2 Center for Translational Cardiology and Pragmatic Randomized Trials, Department of Biomedical Sciences, Faculty of Health and Medical Sciences, University of Copenhagen, 2200 Copenhagen, Denmark; 3 Department of Cardiology, Copenhagen University Hospital—Rigshospitalet, 2100 Copenhagen, Denmark; 4 Steno Diabetes Center Copenhagen, 2730 Herlev, Denmark

**Keywords:** right ventricle, strain, speckle tracking, arrhythmogenic cardiomyopathy, ARVC

## Abstract

Arrhythmogenic right ventricular cardiomyopathy is an inherited cardiomyopathy, characterized by abnormal cell adhesions, disrupted intercellular signaling, and fibrofatty replacement of the myocardium. These changes serve as a substrate for ventricular arrhythmias, placing patients at risk of sudden cardiac death, even in the early stages of the disease. Current echocardiographic criteria for diagnosing arrhythmogenic right ventricular cardiomyopathy lack sensitivity, but novel markers of cardiac deformation are not subject to the same technical limitations as current guideline-recommended measures. Measuring cardiac deformation using speckle tracking allows for meticulous quantification of global systolic function, regional function, and dyssynchronous contraction. Consequently, speckle tracking to quantify myocardial strain could potentially be useful in the diagnostic process for the determination of disease progression and to assist risk stratification for ventricular arrhythmias and sudden cardiac death. This narrative review provides an overview of the potential use of different myocardial right ventricular strain measures for characterizing right ventricular dysfunction in arrhythmogenic right ventricular cardiomyopathy and its utility in assessing the risk of ventricular arrhythmias.

## 1. Introduction

Arrhythmogenic cardiomyopathy is an inherited cardiomyopathy that is most commonly caused by an autosomal dominant mutation and has a prevalence of approximately 1:5000. Since its initial description in 1982 [1], it has widely been recognized as a cardiomyopathy affecting mainly the right ventricle (RV). Even though it most frequently presents as a predominant RV cardiomyopathy, both biventricular and isolated left ventricular (LV) phenotypes have been described. Consequently, arrhythmogenic cardiomyopathy has been proposed as a more accurate term than arrhythmogenic right ventricular cardiomyopathy (ARVC) or dysplasia [2,3]. The term ARVC will, however, be utilized throughout this review for ease of understanding.

Most frequently, genetic mutations affect desmosomal proteins, which are responsible for cell adhesions, resulting in the detachment of cardiomyocytes and disrupted intercellular signaling. In addition, the condition is characterized by fibrofatty infiltrations as replacements of cardiomyocytes, typically located in the triangle of dysplasia: the subtricuspid region, infundibular region, and the apex [4]. These anatomical changes not only impair normal contractility, predisposing to heart failure, but also create a substrate for malignant arrhythmias to develop. 

The 2023 ESC guidelines recommend ECG and cardiac imaging (including echocardiography and/or cardiac magnetic resonance, CMR) in first-degree relatives along with genetic cascade screening [5]. In patients with established cardiomyopathy, echocardiographic evaluation is recommended regularly, i.e., every 1–2 years. Similarly, echocardiography is an essential part of the long-term follow-up strategy in mutation carriers who are phenotype-negative, since the penetrance of the disease may become apparent later in life. Consequently, echocardiography has an important place in the diagnostic process and continuous follow-up in ARVC. 

The 2010 Task Force Criteria (TFC) have been proposed as a tool to support the diagnosis of ARVC [6], and since then, the Padua criteria were proposed in 2020 to optimize the diagnosis of phenotypes with LV involvement [7]. 

The 2010 TFC outline major and minor echocardiographic criteria that include the presence of regional RV akinesia, dyskinesia, or aneurysm in addition to RVOT dilatation or reduced FAC. Even though patients may present with obvious signs of RV abnormalities suggestive of ARVC, some patients may have ‘early’ or ‘subclinical’ ARVC, defined as asymptomatic, genotype-positive patients with no or only partial fulfillment of the TFC. In addition, only 40% of genotype-positive relatives fulfill the TFC at initial evaluation, either due to low penetrance or because RV structural and/or functional abnormalities may not have developed at the time of initial screening [8]. It is, however, well-established that the TFC echocardiographic criteria lack sensitivity for detecting RV abnormalities in ARVC [9,10]. Therefore, identifying novel echocardiographic parameters that are more sensitive than the currently used criteria may aid the diagnostic process in several ways, which is important since arrhythmic events may develop in the absence of obvious structural abnormalities [11].

Since ARVC is still considered a predominantly RV disease, optimizing echocardiographic assessment of RV structure and function remains relevant. In this review, we have sought to provide an overview of the potential utility of myocardial deformation imaging in the context of ARVC. It is worth noting that even though studies have emerged to suggest a potential use of LV strain measures in ARVC [12,13,14], this review focuses on findings related to RV strain obtained using two-dimensional speckle tracking echocardiography (STE).

## 2. Right Ventricular Function

### 2.1. Echocardiographic Assessment of the Right Ventricle

The RV has historically been a challenge to assess using transthoracic echocardiography due to its close-wall anterior location, its complex anatomy, and its intricate contraction pattern. Current echocardiography guidelines set forth by the American Society of Echocardiography and European Association of Cardiovascular Imaging recommend the use of tricuspid annular plane systolic excursion (TAPSE), RV S’ from myocardial tissue Doppler imaging, RV index of myocardial performance (RIMP), and fractional area change (FAC) to assess RV function [15], and similar measures have been highlighted by the British Society of Echocardiography’s practical guidelines for right heart assessment [16]. Of these parameters, however, only the FAC is included in the ARVC TFC. 

### 2.2. Right Ventricular Strain

**Figure 1:**
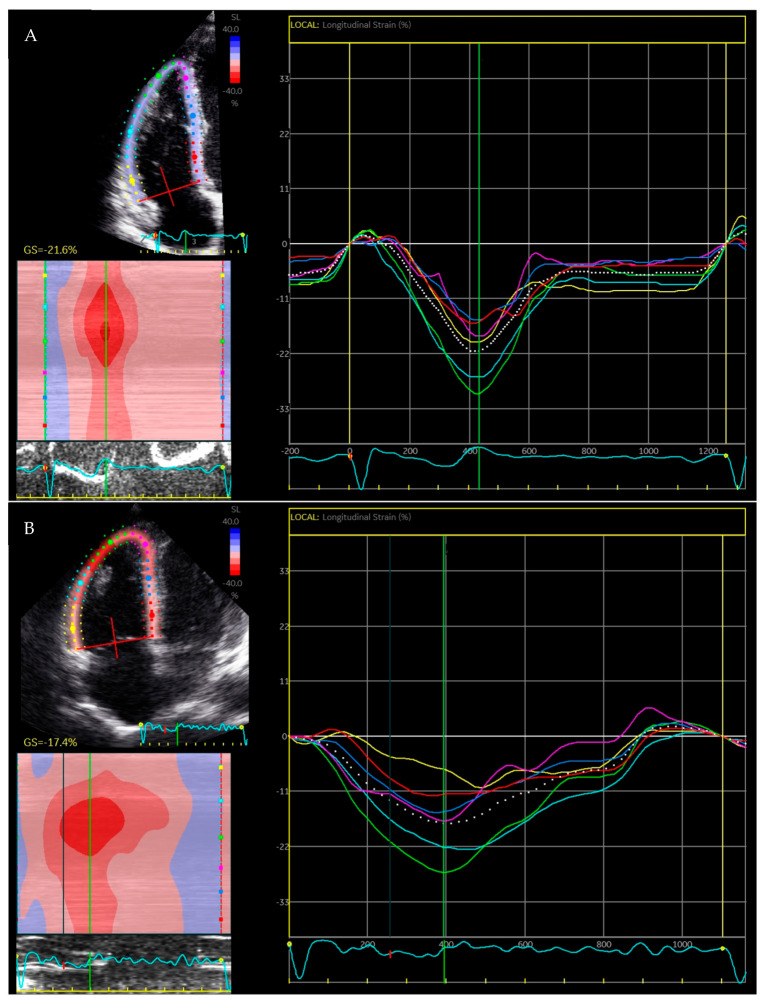
Representative examples of RV speckle tracking. Both figures represent speckle tracking performed in the modified 4-chamber, RV-focused view. (**A**) is an example of a healthy individual. Segmental strain curves are shown as colored lines, whereas the global value is shown as white dotted line. Note the global strain value of −21.6% and the synchronous contraction pattern with peak strain values aligned at the green line (corresponding to pulmonic valve closure). (**B**) is an example of speckle tracking in a patient with overt ARVC. Note the lower global strain value of −17.4%, the heterogenous contraction pattern, and delayed onset of contraction with post-systolic shortening in the subctricuspid segment (yellow curve). RV: right ventricular; ARVC: arrhythmogenic right ventricular cardiomyopathy.

Even though the above-mentioned parameters are recommended in the current guidelines, they all suffer from a specific set of limitations, including load dependency, chest wall deformities, myocardial tethering, and angle dependency [17,18]. Accordingly, novel techniques that are less affected by such factors are warranted. Myocardial deformation imaging through STE offers the opportunity to assess Lagrangian strain, which quantifies the deformation throughout the cardiac cycle against a single reference length from the onset of deformation [19,20]. RV STE therefore introduces a means to quantify both global and regional longitudinal function through absolute strain values in a time-efficient manner of approximately 1 min [21]. In addition, it also offers additional insights into RV mechanics by measuring dyssynchrony (most commonly as mechanical dispersion; MD) and deformation patterns such as post-systolic shortening [22]. Representative examples of RV myocardial deformation analysis in a healthy subject and a patient with overt ARVC are shown in Figure 1A,B.

It is worth noting that RV strain has been assessed differently across studies, either as an estimate from a three-segment model (RV free wall strain; RV FWS) or a six-segment model that includes the interventricular septum (RV global longitudinal strain; RV GLS). RV MD reflects contraction heterogeneity, quantified as the standard deviation of time-to-peak strain, typically from the six-segment model. 

Certain limitations are worth noting for RV STE. Firstly, only longitudinal function is assessed. In addition, strain measures are affected by loading conditions and electro-mechanical activation. Finally, a lack of normative data, intervendor variability, and a lack of dedicated software have been the main reasons for not recommending RV strain for clinical use. 

Even though normative data are still sparse or lacking for regional RV strain and RV MD, they have now become available for RV FWS and RV GLS based on large-scale prospective studies [23], multicenter studies [24], and meta-analyses [25,26].

Table 1 outlines the largest studies reporting normal values for RV strain measurement. Of note, among these studies, only the WASE study and the HUNT study used dedicated RV strain software (Image Arena; TomTech, Unterschleissheim, Germany for the WASE study; EchoPAC SWO version 204, GE Healthcare, Horten, Norway for the HUNT study).

While most studies have applied non-dedicated software developed for LV analysis to quantify RV strain, dedicated software algorithms have now become available. Recently, a comparison of dedicated vs. non-dedicated RV strain software did not find a statistically significant difference in estimating RV strain between the two algorithms [21]. Even though the study also showed similar interrater variability for the two algorithms, with a mean difference of 1.5 to 2.9 in absolute percentage points, future optimization of dedicated software tools could potentially increase reproducibility. Recommendations have also been published to guide the practical approach for performing RV STE, which could further improve dissemination as well as reproducibility [31]. Accordingly, substantial efforts have been made since the latest echocardiography guideline update to make RV STE more applicable for clinical use [32,33].

## 3. Right Ventricular Strain to Characterize ARVC

### 3.1. RV Global Strain

The most widely investigated RV strain parameters have been RV FWS and RV GLS, with several studies showing that these measures have a higher sensitivity for detecting abnormal RV systolic function than conventional measures, including FAC, in populations other than ARVC [34]. This likely reflects that RV strain is a direct measure of myocardial tissue function, as opposed to FAC and TAPSE. Compared with s’ and RIMP, RV strain is not affected by myocardial tethering and angle of insonation. 

Reduced RV strain in patients with overt ARVC compared with healthy controls has been reported [35,36,37,38,39,40,41,42]. These studies are outlined in Table 2. This was also highlighted in a meta-analysis by Qasem et al., which included 10 studies comprising 311 subjects with RV strain analysis (*n*: 154 ARVC patients, *n*: 157 controls), showing mean RV strain values of −17 vs. −29% for ARVC patients vs. controls, respectively, with an optimal cut-off of −21% for distinguishing patients from controls [43]. However, studies have also sought to outline whether patients with subclinical ARVC would also exhibit lower RV strain values. For instance, a prospective study of 70 patients (14 asymptomatic first-degree relatives and 56 age-matched healthy controls) found no differences in RV dimension, FAC, or RVOT, but did show significantly reduced RV FWS in asymptomatic ARVC patients compared with healthy controls (−25% vs. −29%), with 50% of patients having abnormal RV FWS (defined as below 18%) [38]. It is important to bear in mind that ARVC is heterogeneous, in the sense that different mutations lead to different disease expressions, which influences study findings and comparability. In addition, exercise is another confounding factor. Studies have shown that both patients with overt ARVC and mutation carriers who are athletes exhibit reduced biventricular systolic function compared to non-athletes [44]. This is a notable finding, considering the well-established increased VA risk associated with exercise in ARVC [45].

Apart from the above-mentioned case–control studies, studies have also sought to investigate whether STE could inform on disease progression in ARVC. A retrospective study by Malik et al. investigated serial echocardiograms in 40 ARVC patients to evaluate whether functional measures could predict the progression of structural or functional abnormalities, defined as an increase in RVOT diameter or decrease in FAC, respectively [46]. Interestingly, even though RVOT diameter increased, progression was not discernable by FAC. Overall, the authors found that a baseline RV FWS < 20% was associated with a higher likelihood of structural disease progression over a 5-year follow-up. 

### 3.2. Regional Strain

Regional strain has gained particular interest in ARVC for several reasons. Firstly, CMR studies have shown that a quantitative assessment holds higher sensitivity and specificity for detecting regional RV abnormalities than a qualitative assessment in ARVC [47]. Since echocardiographic strain imaging allows for a meticulous quantification of RV mechanics, it could yield higher diagnostic potential than qualitative assessments of regional dysfunction. Secondly, CMR-based studies have also shown that segments with scar tissue, assessed by both late gadolinium enhancement and electroanatomical mapping, exhibit reduced regional strain [48]. Accordingly, regional strain may be a potential marker of regional affliction which may be missed when only considering global values, as with RV FWS or RV GLS. Finally, as previously noted, fibrofatty infiltrations occur more frequently in certain areas of the RV, in particular the subtricuspid region. Accordingly, echocardiographic assessment of basal FWS (corresponding to the subtricuspid region) has been extensively studied as a potential early marker of disease manifestation. Another potential method of evaluating ‘regional’ strain in ARVC is by considering layer-specific strain, since myocyte loss and fibrofatty replacement often develop segmentally [49], starting from the epicardium or the mid-myocardium and extending intramurally to the subendocardium [50,51]. However, this has yet to be explored in dedicated studies. 

In line with the above-mentioned considerations, several case–control studies have shown that patients with both overt and early ARVC exhibit reduced strain in the subtricuspid region as compared with healthy controls [37,52]. However, as previously noted, limited data exist on normative values for regional RV strain. 

**Figure 2:**
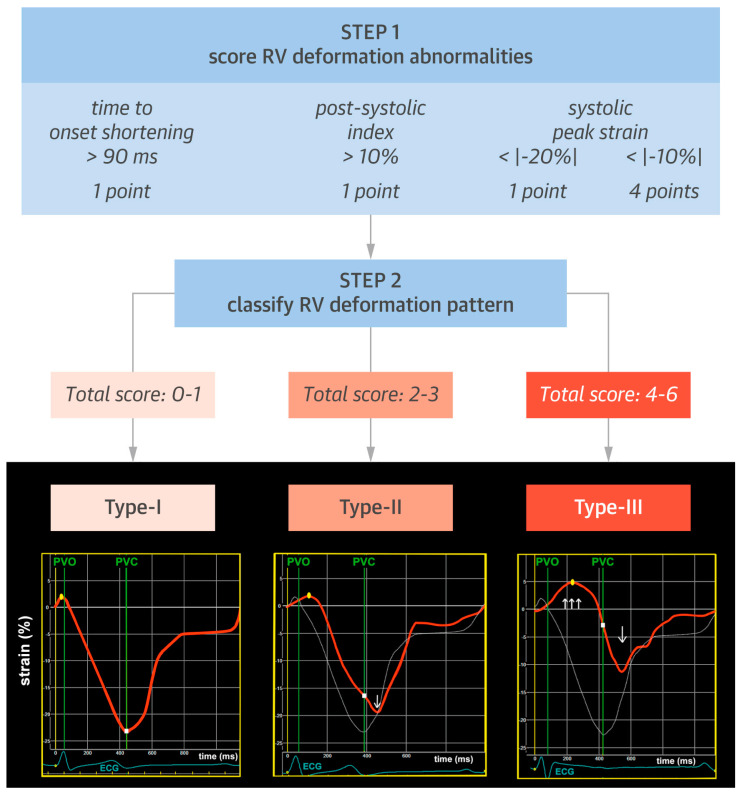
RV deformation pattern classification. Based on the presence of different strain abnormalities in the right ventricular (RV) basal area, 3 distinct characteristic RV deformation patterns were identified: type I represents normal deformation; type II shows delayed onset of shortening (yellow dot) and reduced systolic peak strain (white square) compared with normal deformation and presence of post-systolic shortening (arrow); and type III displays prominent systolic stretching (upward arrows) and passive recoil or shortening (downward arrow) during early diastole. Dotted white lines indicate type I deformation pattern for comparison. PVO/PVC 1⁄4 pulmonary valve opening/closure” Mast et al. [52]. Reproduced with permission from RightsLink/Elsevier.

Recently, a growing interest has been directed towards deformation pattern recognition in the subtricuspid region. In a retrospective study, Mast et al. evaluated RV deformation patterns using STE performed in the subtricuspid region in ARVC patients and controls (total sample *n* = 168, matched 1:1) [52]. The authors identified three distinctive patterns of RV deformation, characterized by the degree of systolic stretching, post-systolic shortening, and peak systolic strain, which emerged after comparing healthy controls with subclinical ARVC patients (no TFC fulfilled), electrical stage patients (rhythm criteria fulfilled), and mechanical ARVC (imaging criteria fulfilled). The deformation patterns are shown in Figure 2. The controls almost exclusively exhibited a normal deformation pattern (type I), whereas half of those with subclinical ARVC exhibited an abnormal (type II) pattern, and nearly all in the electrical stage had either an abnormal type II or III pattern. Overall, a more pathological deformational pattern was increasingly frequent with higher extent of clinical disease expression. Subsequent simulations revealed that a combination of reduced contractility and increased passive stiffness primarily explained the occurrence of these abnormal deformation patterns, as opposed to an electrical substrate defined by an electromechanical delay. These findings demonstrate that genotype-positive patients who do not fulfill imaging TFC may still exhibit abnormal RV mechanics and that mechanical alterations do occur even in the electrical stage of ARVC. This underlines that RV strain could provide sensitive information preceding abnormalities detected using conventional TFC. In addition, the reproducibility of detecting these patterns was excellent, potentially making them more clinically applicable than absolute strain values.

### 3.3. Mechanical Dispersion

MD was initially introduced as a tool to quantify contraction heterogeneity in the LV that could predict ventricular arrhythmias (VAs) in patients with long QT syndrome and patients with myocardial infarction [53,54]. RV MD has been proposed as a marker of VAs in patients with ARVC since it may reflect electrical dispersion (thought to be the primary cause of VAs in the electrical stage). In addition, RV MD may reflect fibrofatty infiltration and scar burden (similar to what has been described for the LV) that poses a risk of VAs in the later stages of ARVC. This notion has been supported in a study from 2011 by Sarvari et al., showing that patients with overt ARVC (*n* = 42) have higher RV MD than both patients with early ARVC and healthy controls (52 ms vs. 35 ms vs. 13 ms, *p* < 0.001) [12]. Interestingly, those with early ARVC also had higher RV MD compared with controls. The authors further noted a high reproducibility, albeit lower than RV GLS, with interclass correlation coefficients (ICCs) of 0.84 for RV MD. A notable finding made in another study by the same research group was that RV MD was more pronounced in early ARVC patients compared with patients with VT originating from the RVOT (22 ± 15 ms vs. 15 ± 13 ms, *p* = 0.03), further emphasizing pathological contraction heterogeneity in early ARVC [55]. 

## 4. Right Ventricular Strain and Ventricular Arrhythmia

Patients with ARVC are at increased risk of VAs, even in the early stages of the disease. Consequently, ARVC is one of the most frequent causes of sudden cardiac death. Determining the risk of VAs is indicated to determine eligibility for implantation of a primary prevention implantable cardioverter defibrillator (ICD) to prevent sudden cardiac death. Although risk algorithms have been devised to support the decision [56], the risk assessment remains elusive, which is also emphasized by current guidelines [5,57]. 

Accordingly, several studies have investigated whether RV strain measures could represent sensitive markers of VA risk in patients with ARVC. An outline of these studies is shown in Table 3.

The earliest study relating RV myocardial deformation characteristics to VAs was conducted by Sarvari et al. in 2011 [12]. The authors measured both LV GLS, LV MD, RV FWS, and RV MD in 69 ARVC patients (*n* = 42 with symptomatic ARVC and history of VT or VF and *n* = 27 asymptomatic mutation carriers) and compared these with both healthy controls (*n* = 30) and with genotype-negative individuals who were relatives to patients with ARVC (*n* = 10). The authors observed that patients with overt ARVC exhibited the lowest LV GLS (−17% vs. −20% vs. −22% for overt ARVC, asymptomatic ARVC, and controls, respectively). Similarly, they exhibited the absolute lowest RV FWS (−19% vs. −22% vs. −25%), highest RV MD (52 ms vs. 35 ms vs. 13 ms), and highest LV MD (60 ms vs. 38 ms vs. 20 ms). Furthermore, patients with asymptomatic ARVC had lower LV GLS and RV FWS and higher LV and RV MD compared with healthy controls, whereas no differences were observed in conventional RV measures. Of all measures, only FAC and RV MD were independently associated with VAs, with RV MD showing the highest area under the curve with an optimal cut-off of 29 ms. The findings highlighted subclinical biventricular involvement in patients with early ARVC and underlined the potential of RV MD as an arrhythmia marker. 

These findings have since been extended in a retrospective study of 162 ARVC patients (*n* = 89 with overt ARVC, *n* = 73 with early ARVC) by Leren et al. [59], who investigated biventricular deformation characteristics (LV and RV GLS and LV and RV MD) in relation to history of VAs (defined as any VT, aborted cardiac arrest, and syncope of suspected cardiac cause). The authors observed that patients with a history of VAs (*n* = 84, 69 with overt ARVC, 15 with early ARVC) had abnormal deformation characteristics by all accounts. In those with overt ARVC, RV GLS were associated with VAs, which was not the case among those with early ARVC, in whom RV MD seemed to be a more sensitive parameter of VAs and provided incremental predictive value to electrical parameters from signal-averaged ECGs. These findings show that these RV strain measures may also be of potential value in early stages of ARVC. In addition, they show a potential differential association between RV strain measures and VAs depending on whether patients have early or overt ARVC. Finally, the findings were made more clinically relevant as the authors examined whether these measures added value on top of other clinical tools, in this case, signal-averaged ECGs. 

Since the endpoint in these studies was defined as a history of VAs, a notable limitation for both studies was the lack of prospective data linking RV GLS and MD to VAs. The first prospective study was performed by Lie et al. [14], who included 117 ARVC patients (67% definite ARVC, 29% probands, 71% genotype-positive relatives) and investigated how clinical characteristics, ECG features, and imaging parameters related to VAs (defined as sustained VT, aborted cardiac arrest, and appropriate ICD therapy). Echocardiographic strain parameters included both LV GLS, LV MD, RV FWS, and RV MD. Of any echocardiographic parameters, only RV FWS and LV MD were independently associated with incident first-time VAs (*n* = 18, median follow-up of 2.0 years), with RV FWS < 23% conferring an increased risk of VAs. Notably, LV MD yielded the highest discrimination for predicting VAs using C-statistics, underlining the importance of also looking at myocardial deformation characteristics in the LV. 

Following the study by Mast et al., who proposed to look at deformation patterns in the subtricuspid region, Kirkels et al. performed a retrospective study to evaluate whether such deformation patterns could be used to indicate VA risk [61]. Based on 160 patients composed of both probands and genotype-positive relatives, Kirkels et al. evaluated the association between RV strain measures and history of VAs (defined as sustained VT, aborted cardiac arrest, and appropriate ICD therapy). Of the 160 patients, 61% had definite ARVC. In this study, 98% of patients who had a history of VAs had an abnormal type II or III deformation pattern. Every increment in deformation pattern was independently associated with VAs. RV MD was also independently associated with VAs, and combining these two strain features improved discrimination for predicting VAs. A notable finding was the low specificity of the deformation patterns but high negative predictive value of 98% with a normal deformation pattern. The same authors built on these findings in another prospective cohort study of 150 patients with overt ARVC and no prior history of VAs. Here, Kirkels et al. investigated the value of RV FWS, RV MD, and RV deformation patterns for predicting VAs [60]. During a median follow-up of 6.3 years (IQR: 3.1–9.8), 37 patients developed VA, defined as sustained VT, aborted cardiac arrest, or appropriate ICD therapy. As opposed to prior studies, the novelty of this study was that it evaluated whether these RV strain measures added value on top of the clinically used ARVC risk calculator. The authors observed that all RV strain measures were independently associated with VAs and that increasingly abnormal RV FWS and abnormal deformation patterns were associated with a higher risk of VAs in an incremental fashion. Finally, both RV FWS and abnormal deformation patterns increased discrimination for predicting VAs. In addition, they again confirmed the high negative predictive value of 96% with a normal type I deformation pattern, underlining that deformation patterns may be the most valuable measure to identify patients at low risk of VAs. 

Overall, the findings of the studies are consistent in the sense that myocardial deformation measures in general may provide early markers of VA risk and in showing that ARVC patients exhibit abnormalities in global biventricular strain and MD. However, the optimal measure for risk stratification in these patients is not as consistent. This may be due to the heterogenous samples, relatively small sample sizes due to the inherent rarity of ARVC, study designs, and definition of the VA endpoint. Although one prospective study found LV MD to be the strongest marker of VA risk [14], the recent finding by Kirkels et al. did not find that this measure added value on top of the ARVC risk calculator [60]. As such, RV FWS and deformation patterns in the subtricuspid region seem the most promising markers of VA risk, particularly in patients with overt ARVC, although the findings need to be validated externally.

## 5. Conclusions

The use of two-dimensional speckle-tracking echocardiography allows for the identification of minuscule abnormalities in myocardial mechanics. These abnormalities seem to indicate abnormal RV systolic function and dyssynchrony in early stages of ARVC, even in patients who do not fulfill ARVC Task Force Criteria but are genotype-positive.

In addition, RV strain may potentially be used to identify patients likely to develop structural RV abnormalities. Several deformation measures of RV function have been linked to an increased risk of ventricular arrhythmias; however, the studies have been relatively minor and heterogeneous in design, challenging direct comparisons. In patients with overt ARVC, particularly regional deformation patterns and abnormal free wall strain seem to add prognostic information beyond clinical risk tools concerning the risk of ventricular arrhythmias and sudden cardiac death. However, additional large-scale prospective studies are still needed for external validation and to further substantiate the potential use of RV strain measures. 

## Figures and Tables

**Table 1:** Lower limit of normality for RV strain measures.

	CCHS [23] *n*: 1297	WASE [24] *n*: 1913	Muraru et al. [27] *n*: 276	NORMAL [28] *n*: 493	HUNT [29] *n*: 1103	Fine et al. [30] ** *n*: 489	Wang et al. [26] ** *n*: 3673
RV FWS	−16.5%	−20.0%	−23.3%	−18.0%	−17.4%	−24.0%	−18.0%
RV GLS	−15.0%	−18.2%	−20.2%	15.1%	-	-	−16.4% *
RV apical strain	-	-	−14.9%	-	-	-	-
RV mid strain	-	-	−22.3%	-	-	-	-
RV basal strain	-	-	−20.0%	-	-	-	-
RV MD	-	-	-	-	-	-	-

CCHS: Copenhagen Heart Study; WASE: World Alliance Society of Echocardiography; NORMAL: Normal EchOcardiogRaphic diMensions and function in KoreAn PopuLation; HUNT: Helseundersøkelsen i Trøndelag; RV: right ventricular; FWS: free wall strain; GLS: global longitudinal strain; MD: mechanical dispersion. * available in 2674 subjects ** meta-analysis.

**Table 2:** Mean value of RV strain in subjects with ARVC compared to healthy individuals.

Study	Population	RV FWS (%)	RV GLS (%)	RV Basal Strain (%)	RV Mid Strain (%)	RV Apical Strain (%)	Proposed Cut-Off **	AUC **	Sensitivity (%) **	Specificity (%) **
Teske et al. [35]	34 D-ARVC	−17.8 †	-	−11.1 †	−16.5 †	−19.0 †	RV FWS: −25.1	0.96	90	100
	34 healthy controls	−29.6 †	-	−25.4 †	−29.7 †	−31.9 †				
Pieles et al. [36]	38 D-ARVC	−19.0 †	−21.0 †	−21.0 †	−19.0 †	−17.0 †	RV GLS: −20.4	0.84	53	100
	39 B-ARVC	−21.0 †	−23.0 †	−24.0 †	−21.0 †	−19.0 †				
	43 P-ARVC	−24.0 †	−26.0 †	−28.0 †	−23.0 †	−22.0 †				
	35 healthy controls	−24.0 †	−25.0 †	−27.0 †	−23.0 †	−20.0 †				
Vitarelli et al. [37]	19 D-ARVC	-	−20.4 †	-	-	-	RV GLS: −25.0	0.86	85	90
	19 healthy controls	-	−28.6 †	-	-	-				
Teske et al. [38]	14 AM-ARVC	−25.0 †	-	−19.5 †	−23.8 †	−28.4	RV FWS: −18.0	-	71	81
	56 healthy controls	−29.0 †	-	−25.2 †	−28.2 †	−31.2				
Prakasa et al. [39] *	30 D-ARVC	−10.0 †	-	-	-	-	RV FWS: −18.0	0.82	73	87
	36 healthy controls	−28.0 †	-	-	-	-				
Wang et al. [40] *	10 D-ARVC	−17.2 †	-	-	-	-	-	-	-	-
	43 healthy controls	−33.3 †	-	-	-	-				
Tops et al. [41] *	52 D-ARVC	−19.0 †	-	-	-	-	-	-	-	-
	25 healthy controls	−25.0 †	-	-	-	-				
Iacoviello et al. [42]	15 D-ARVC	−25.6 †	-	−25.1 †	−27.5 †	−24.1	-	-	-	-
	25 healthy controls	−31.4 †	-	−32.7 †	−34.0 †	−27.3				

Abbreviation: RV: right ventricular; FWS: free wall strain; GLS: global longitudinal strain; AUC: area under the curve; D-ARVC: definite arrhythmogenic right ventricular cardiomyopathy; B-ARVC: borderline arrhythmogenic right ventricular cardiomyopathy; P-ARVC: possible arrhythmogenic right ventricular cardiomyopathy; AM-ARVC: asymptomatic mutation positive first-degree relatives of ARVC probands. Proposed cut-off, AUC, sensitivity, and specificity refer to the values for distinguishing ARVC from controls. * Determined RV strain from tissue Doppler imaging. ** Refers to either RV FWS or RV GLS; regional strain not included. † Denotes significant differences between cases and controls. Negative and positive predictive values are not specified in any of the studies.

**Table 3:** Studies examining the association between RV strain measures and ventricular arrhythmias.

Study	Year	Design	Sample	Outcome	Arrhythmia Detection	Events	Follow-Up (Years)	Key Findings
Sarvari et al. [12]	2011	Prospective inclusion Retrospective endpoints Case–control study	69 ARVC, 40 controls - 42 probands - 27 asymptomatic, genotype-positive relatives - 30 healthy controls - 10 genotype-negative, healthy relatives,	History of either: VT VF	Holter (asymptomatic ARVC patients)	42	N/A	RV MD and FAC were independently associated with a history of VA.
Alizade et al. [58]	2016	Cross-sectional study Retrospective endpoints	34 definite ARVC - 17 asymptomatic - 17 symptomatic	History of: Sudden cardiac arrest VT	Holter (symptomatic ARVC patients)	17	N/A	RV FWS was significantly reduced in patients with a history of VA.
Leren et al. [59]	2017	Cross-sectional study Retrospective endpoints	162 overall - 55% definite ARVC - 53% probands	History of either: Any VT ACA Cardiac syncope	Holter ICD Exercise test	84	N/A	In patients with definite ARVC, RV GLS was associated with VAs. In patients with early ARVC, RV MD was associated with VAs.
Lie et al. [14]	2018	Prospective	117 overall - 56% definite ARVC - 29% probands - 71% genotype-positive relatives	Composite: Sustained VT Cardiac arrest Appropriate ICD shock	ECG Holter ICD	18	2.0 (IQR: 0.5–3.5)	RV FWS and LV MD were independently associated with VAs.
Kirkels et al. [60]	2021	Cross-sectional study Retrospective endpoints	160 overall - 61% definite ARVC - 43% probands - 57% relatives	History of either: Sustained VT Appropriate ICD therapy ACA	ECG Holter ICD	47	N/A	RV MD and subtricuspid deformation pattern were independently associated with history of VAs.
Kirkels et al. [61]	2023	Prospective	150 definite ARVC - 34% probands - 66% relatives	Composite: Sustained VT ACA Appropriate ICD therapy	Not specified	37	6.3 (IQR: 3.1–9.8)	LV GLS, RV FWS, and RV MD were strongest predictors of VAs. Only RV FWS and MD improved discrimination on top of the ARVC risk calculator.

ARVC: arrhythmogenic right ventricular cardiomyopathy; IQR: interquartile range; LV: left ventricular; RV: right ventricular; GLS: global longitudinal strain; MD: mechanical dispersion; FWS: free wall strain; VA: ventricular arrhythmia; VT: ventricular tachycardia; VF: ventricular fibrillation; ICD: implantable cardioverter defibrillator; ACA: aborted cardiac arrest.

## Data Availability

Not applicable.
